# Systemic Immune Dysregulation Correlates With Clinical Features of Early Non-Small Cell Lung Cancer

**DOI:** 10.3389/fimmu.2021.754138

**Published:** 2022-01-18

**Authors:** Zhixing Hao, Mingjie Lin, Feng Du, Zhongwei Xin, Dang Wu, Qun Yu, Yimin Wu, Zhouyu Zhu, Wenshan Li, Yongyuan Chen, Xiaoke Chen, Ying Chai, Shenghang Jin, Pin Wu

**Affiliations:** ^1^ Department of Thoracic Surgery, The Second Affiliated Hospital, Zhejiang University School of Medicine, Zhejiang University, Hangzhou, China; ^2^ Key Laboratory of Tumor Microenvironment and Immune Therapy of Zhejiang Province, The Second Affiliated Hospital, Zhejiang University School of Medicine, Zhejiang University, Hangzhou, China; ^3^ Department of Thoracic Surgery, Yuhang Branch of The Second Affiliated Hospital, Zhejiang University School of Medicine, Zhejiang University, Hangzhou, China; ^4^ Department of Oncology Radiotherapy, The Second Affiliated Hospital, Zhejiang University School of Medicine, Zhejiang University, Hangzhou, China; ^5^ Fourth Ward of Neurosurgery, Division of Nursing, The Second Affiliated Hospital, Zhejiang University School of Medicine, Zhejiang University, Hangzhou, China; ^6^ Department of Clinical Laboratory, The Second Affiliated Hospital, Zhejiang University School of Medicine, Zhejiang University, Hangzhou, China

**Keywords:** peripheral blood, non-small cell lung cancer, lymphocyte cells, systemic immune dysregulation, systemic inflammatory response syndrome

## Abstract

**Background:**

Systemic immune dysregulation correlates with cancer progression. However, the clinical implications of systemic immune dysregulation in early non-small cell lung cancer (NSCLC) remain unclear.

**Methods:**

Using a panel of 9 markers to identify 12 parameters in the peripheral blood of 326 patients (34 in the discovery group and 292 in the validation group), we investigated systemic immune dysregulation in early NSCLC. Then, we analyzed the impact of surgery on the systemic immune state of these patients. Finally, we analyzed correlations between systemic immune dysregulation and the clinical features of early NSCLC.

**Results:**

We found striking systemic immune dysregulation in the peripheral blood of early NSCLC patients. This dysregulation was characterized by a significant decrease in total lymphocytes, T cells, quiescent T cells, CD4+ T cells, and NKT cells. We also observed increased proportions of activated lymphocytes and activated T cells. Systemic immune dysregulation was increased after surgery. Furthermore, systemic immune dysregulation was correlated with multiple clinical features, such as sex, age, smoking history, pathological type, tumor stage, surgical approach, tumor differentiation, and epidermal growth factor receptor (EGFR) mutation. Finally, we observed that systemic immune dysregulation was correlated with complications and systemic inflammatory response syndrome (SIRS) in early NSCLC patients.

**Conclusions:**

Our results reveal systemic immune dysregulation occurring in early NSCLC and demonstrate the correlation between these dysregulations and clinical features. Our findings suggest that systemic immune dysregulation is involved in cancer development and may be a promising candidate for high-risk screening and treatment strategies for early NSCLC.

## Introduction

Non-small cell lung cancer (NSCLC) is a common cancer type that leads to morbidity and mortality worldwide (1). In China, the incidence and fatality rate of NSCLC are at the top of the list and show increasing prevalence in recent years (2). Despite advancements in NSCLC treatment that have been achieved over the past two decades, the overall survival (OS) rates for NSCLC remain low (3). Emerging findings suggest that functional recovery of preexisting dysfunctional immune cells in tumors and the peripheral blood (PB) is key for successful immunotherapy (4). Therefore, further studies aiming to increase our understanding of the immune changes in human NSCLC are needed.

A recent study showed that resident immune cells in pre-invasive lesions became dysfunctional in the early stages of human lung squamous carcinogenesis (5). Another single-cell study showed that the tumor-infiltrating myeloid cell subsets change during the early stages of human lung adenocarcinoma (LUAD) (6). Single-cell RNA sequencing demonstrated that tumor-infiltrating lymphocytes (TILs) have a dysfunctional phenotype in early human NSCLC (7, 8). Another study found that most dysfunctional TILs show resident immune cell features (9, 10). These findings suggest an unexpected phenomenon in which local immune perturbation in the tumor microenvironment occurs as early as during the initiation stage of human NSCLC (11). Whether any immune perturbation arises at the systemic level in NSCLC remains unclear.

Lymphocytes in the PB, which are mainly composed of T, B, NK, and NKT cells, play a crucial role in systemic immune homeostasis. A previous study showed that altered DNA methylation occurs in peripheral leukocytes of patients with small cell lung cancer (SCLC) (12). Further studies showed that DNA methylation changes in pre-diagnostic PB samples were associated with smoking and lung cancer risk (13). Another study showed that telomere dysfunction occurs in the PB leukocytes of NSCLC patients and correlates with cancer risk (14). An enhanced lymphocyte-to-monocyte ratio (LMR) is correlated with better survival of NSCLC patients (15). A prognostic nomogram incorporating clinical data and PB markers, including the pretreatment neutrophil-to-lymphocyte ratio (LMR), shows good accuracy in predicting the OS of SCLC (16, 17). Expression of TNFR2 by regulatory T cells in PB correlates with the clinical pathology of lung cancer patients (18). The PB T-cell receptor (TCR) repertoire correlates with disease development and prognosis in advanced lung cancer (19). These studies suggest that systemic immune dysregulation emerges in the PB of patients with advanced NSCLC and that these dysregulations correlate with the outcome. However, whether systemic immune dysregulation also exists in the PB of early NSCLC remains unknown.

Here, we used a panel of nine markers to profile 12 parameters in the PB of 326 patients with early NSCLC. Then, we investigated systemic immune perturbations in the discovery group and validation group. Our study found an observable change in the immune state of early NSCLC patients with respect to systemic immune dysregulation, which correlated with clinical features such as gender, age, and tumor stage.

## Materials and Methods

### Patients and Healthy Donors

Fresh PB was obtained from NSCLC patients without previous treatment who underwent surgical resection at the Second Affiliated Hospital, Zhejiang University School of Medicine. Control PB samples were obtained from 34 healthy donors, all of whom were negative for antibodies against hepatitis C virus, hepatitis B virus, HIV, and syphilis. A total of 34 patients with non-advanced NSCLC were included in the discovery group, and 292 patients with non-advanced NSCLC were included in the validation group. The main characteristics of the subjects are summarized in **Supplementary Table 1**. All samples were anonymously coded in accordance with local ethical guidelines (as stipulated by the Declaration of Helsinki). Written informed consent was obtained from all study participants. The study protocol was approved by the Review Board of the Second Affiliated Hospital of Zhejiang University School of Medicine.

### Cell Preparations and Flow Cytometry

Antibodies against CD45 (HI30), CD3 (UCHT1), CD4 (RPA-T4), CD8a (RPA-T8), CD38 (HB-7), CD16 (3G8), CD56 (HCD56), and HLA-DR (L243) were purchased from BioLegend. PB lymphocytes were collected after lysing red blood cells with lysing solution (BD Pharm Lyse). To block non-specific binding and to stain with combinations of fluorochrome-coupled antibodies at 4°C for 15 min, we preincubated peripheral blood mononuclear cells (PBMCs) (1 × 10^6^/ml) in phosphate-buffered saline (PBS), 2% fetal bovine serum, and 0.1% (w/v) sodium azide with FcgIII/IIR-specific antibody. Flow cytometry data were collected using a FACSCanto II system and FACSFortessa system (BD Biosciences) and were analyzed using FlowJo software (Tree Star).

### The Definition of Lymphocyte Subsets

The lymphocyte subsets in our study were defined as total lymphocytes (CD45+ SSC-low), activated lymphocytes (CD38+ CD45+), T lymphocytes (CD3+ CD45+), B lymphocytes (CD19+ CD45+), NK cells (CD16+ CD56+ CD3− CD45+), NKT cells (CD16+ CD56+ CD3+ CD45+), T helper cells (CD4+ CD3+ CD45+), T cytotoxic cells (CD8+ CD3+ CD45+), activated T lymphocytes (HLA-DR+ CD3+ CD45+), resting T lymphocytes (HLA-DR− CD3+ CD45+ lymphocytes), and activated T cytotoxic cells (HLA-DR+ CD8+ CD45+) (20). A summary of the immunophenotyping analysis for cancer patients and normal subjects is shown in **Supplementary Table 2**.

### Statistical Analysis

All results are expressed as the mean ± standard error of the mean (SEM). Statistical analysis between healthy people and NSCLC patients was performed using the Mann–Whitney U test. Pairwise comparisons between pre- and post-surgical data were analyzed using the Wilcoxon matched-pairs signed-rank test. Multiple groups were compared using ordinary one-way ANOVA and Tukey’s multiple comparisons test if the data were normally distributed and had uniform variance. Otherwise, the Kruskal–Wallis tests and Dunn’s multiple comparisons tests were used. Correlations were analyzed using Spearman’s rank correlation coefficient. Statistical analyses were performed using GraphPad Prism software version 6.1. Statistical significance was set at *p* < 0.05.

## Results

### Systemic Immune State Is Dramatically Changed in Early Non-Small Cell Lung Cancer

**Figure 1:**
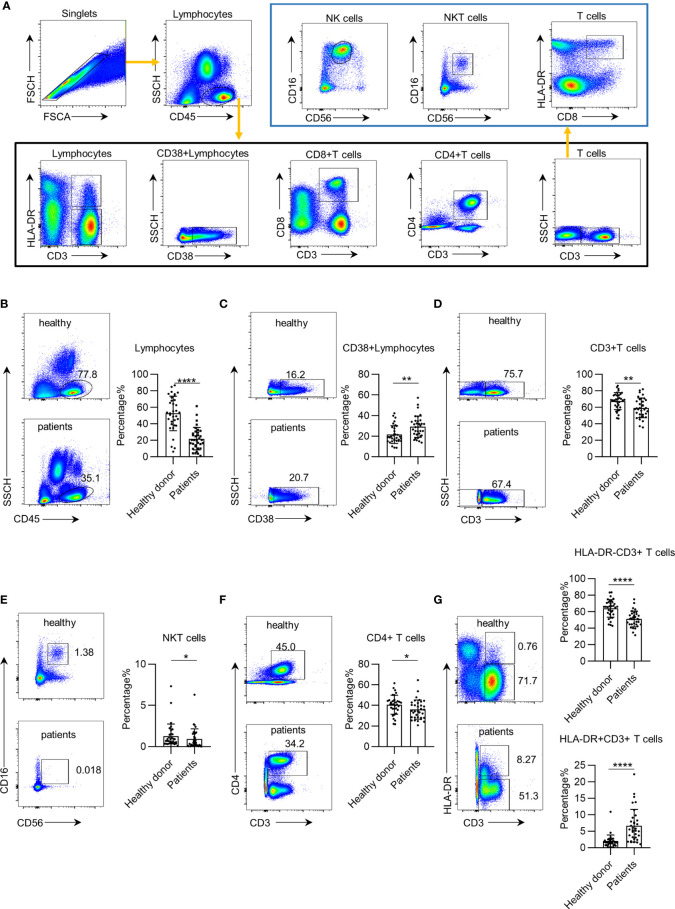
Systemic immune dysregulation in early non-small cell lung cancer (NSCLC). Systemic immune dysregulation was assessed by flow cytometry on single-cell suspensions prepared from the peripheral blood of NSCLC patients and healthy donors. **(A)** Representative flow cytometric analysis of peripheral lymphocyte cells and the gating strategy used in this study. **(B–G)** Representative flow cytometric analysis of total lymphocytes **(B)**, activated lymphocytes **(C)**, CD3+ T cells **(D)**, NKT cells **(E)**, CD4+ T cells **(F)**, and quiescent and activated CD3+ T cells **(G)** in the peripheral blood of NSCLC patients and healthy donors (left). Bar plots summarizing the percentage of total lymphocytes **(B)**, activated lymphocytes **(C)**, CD3+ T cells **(D)**, NKT cells **(E)**, CD4+ T cells **(F)**, and quiescent and activated CD3+ T cells **(G)** in the peripheral blood of NSCLC patients and healthy donors (right). Data are shown as mean ± SEM; NSCLC patients, n = 34; healthy donors, n = 34. *p < 0.05; **p < 0.01; ****p < 0.0001.

To establish an approach to profile leukocyte subsets in the PB of patients with early NSCLC, we used a panel of nine markers to define 11 leukocyte subsets, similar to previous studies (21). The detailed gate strategy is shown in **Figure 1A**. We first profiled the leukocyte subsets in the PB of 34 healthy donors and 34 preoperative patients with early NSCLC who were subsequently pathologically confirmed. The age distribution between patients and controls was statistically similar. Our results showed that the total leukocyte percentages and absolute counts in the PB of preoperative patients with early NSCLC were significantly lower than those in healthy donors (**Figure 1B** and **Supplementary Figure 1A**). In contrast, the percentage of CD38+ CD45+ leukocytes in the PB of preoperative patients was higher than that in healthy donors (**Figure 1C**). However, the absolute counts of CD38+ CD45+ leukocytes showed a decreasing trend in NSCLC, which may be due to the decrease in total leukocytes (**Supplementary Figure 1B**). Furthermore, the number of CD3+ T cells and NKT cells in preoperative patients decreased (**Figures 1D, E** and **Supplementary Figures 1C, D**). Further analysis showed a decreasing trend of HLA-DR− CD3+ T cells and CD4+ T cells in preoperative patients with early NSCLC (**Figures 1F, G** and **Supplementary Figures 1E, F**). However, the percentage of HLA-DR+ CD3+ T cells in preoperative patients with early NSCLC was higher than that in healthy donors (**Figure 1G**). These systemic immune changes were validated using paired samples of patients postoperatively (**Supplementary Figure 2**). These findings demonstrate that systemic immune dysregulation occurs in the PB of patients with early NSCLC.

### Surgery Impacts the Systemic Immune State of Patients

**Figure 2:**
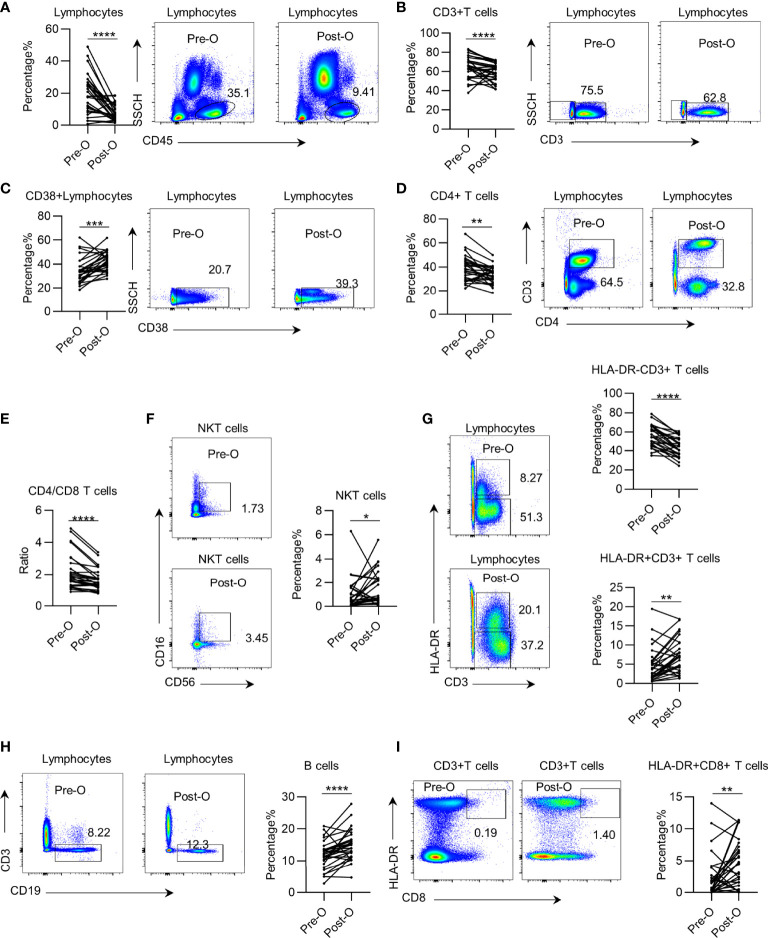
The impact of surgery on systemic immune dysregulation in early non-small cell lung cancer (NSCLC). Systemic immune dysregulation was assessed by flow cytometry on single-cell suspensions prepared from paired pre- and postoperative peripheral blood samples from NSCLC patients. **(A–D)** Representative flow cytometric analysis of total lymphocytes **(A)**, CD3+ T cells **(B)**, activated lymphocytes **(C)**, and CD4+ T cells **(D)** in the paired pre- and postoperative peripheral blood samples from NSCLC patients (left). Bar diagram summarizing the percentage of total lymphocytes **(A)**, CD3+ T cells **(B)**, activated lymphocytes **(C)**, and CD4+ T cells **(D)** before and after surgery (right). Pre-O, preoperative; Post-O, postoperation. Data are shown as mean ± SEM; n = 29; *p < 0.05; **p < 0.01; ***p < 0.001; ****p < 0.0001. **(E)** Bar diagram summarizing the ratio of CD4+ T cell/CD8+ T cells in the peripheral blood of NSCLC patients pre- and postoperatively. Pre-O, preoperative; Post-O, postoperation Data are shown as mean ± SEM; n = 29; ****p < 0.0001. **(F–I)** Representative flow cytometric analysis of NKT cells **(F)**, quiescent and activated CD3+ T cells **(G)**, B cells **(H)**, and activated CD8+ T cells **(I)** in the paired pre- and postoperative peripheral blood samples from NSCLC patients (left). Bar plot summarizing the percentage of NKT cells **(F)**, quiescent and activated CD3+ T cells **(G)**, B cells **(H)**, and activated CD8+ T cells **(I)** before and after surgery (right). Pre-O, preoperative; Post-O, postoperation. Data are shown as mean ± SEM; n = 29; *p < 0.05; **p < 0.01; ***p < 0.001; ****p < 0.0001.

Next, to investigate whether the systemic immune dysregulation in patients with early NSCLC is impacted by surgery, we profiled leukocyte subsets in paired PB samples acquired pre- and postoperatively from 29 patients in the discovery group. We found that after surgery, the trend changed in the same direction as that in the preoperation samples. We found that the total leukocyte and CD3+ T cell percentage and absolute numbers in the postoperative PB of patients were further decreased (**Figures 2A, B** and **Supplementary Figures 3A, B**). CD38+ CD45+ leukocytes showed decreased in postoperative patients. However, the CD38+ CD45+ leukocyte percentage increased due to a total decrease in lymphocytes (**Figure 2C** and **Supplementary Figure 3C**). Further analysis of the T cell subsets showed that the CD4+ T cells and of CD4+/CD8+ T cell ratio significantly decreased in postoperative patients (**Figures 2D, E** and **Supplementary Figures 3D, E**). The percentage of NKT cells and HLA-DR+ CD3+ T cells in postoperative patients significantly increased (**Figures 2F, G**), but this increase was not present in the absolute counts (**Supplementary Figures 3F, G**). Consistently, the percentage and absolute number of HLA-DR− CD3+ T cells significantly decreased in postoperative patients (**Figure 2G** and **Supplementary Figure 3G**). Furthermore, we observed an increase in the percentage of B cells and HLA-DR+ CD8+ T cells (**Figures 2H, I**), which was opposite from the decrease in absolute value due to the decrease of total lymphocytes (**Supplementary Figures 3H, I**). Regarding the surgical procedure, we observed slight differences in systemic immune dysregulation between patients receiving sub-lobectomy and lobectomy (**Supplementary Figure 4**). Together, these findings suggest that the systemic immune state of early NSCLC is further impacted by surgery.

### Systemic Immune Dysregulation Correlates With the Clinical Features of Non-Small Cell Lung Cancer

The clinical features of NSCLC are correlated with treatment and patient outcomes (3). However, the correlation between clinical features and the systemic immune state in NSCLC remains unclear. We analyzed the correlation between clinical features and peripheral leukocyte subsets in another cohort of 292 early NSCLC patients to validate systemic immune dysregulation and to investigate potential correlations with clinical features of NSCLC. All findings in the discovery group were verified in the validation group (**Supplementary Figure 5**).

**Figure 3:**
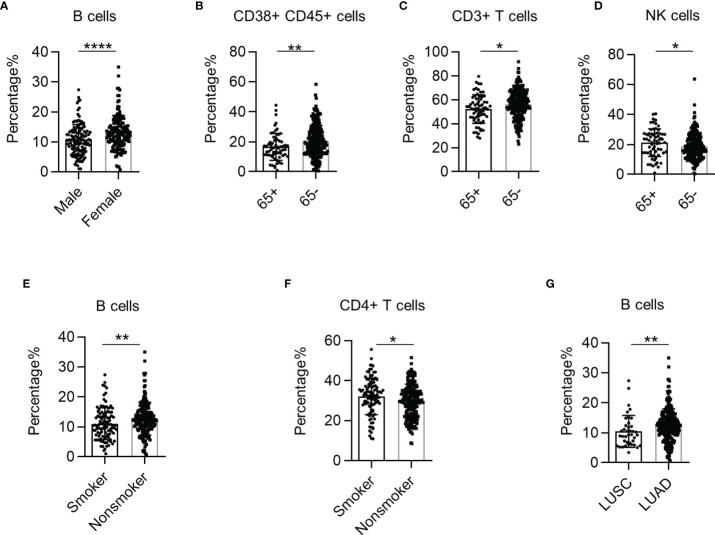
The correlation between systemic immune dysregulation and baseline patient characteristics. Systemic immune dysregulation in the validation group was analyzed to investigate the correlation between systemic immune dysregulation and baseline patient characteristics. **(A)** Bar diagram summarizing the percentage of B cells in the peripheral blood samples from non-small cell lung cancer (NSCLC) patients according to sex. Male, n = 131; female, n = 150. **(B–D)** Bar diagram summarizing the percentage of activated lymphocytes **(B)**, CD3+ T cells **(C)**, and NK cells **(D)** in the peripheral blood of NSCLC patients according to age. 65+, above 65 years old; 65−, under 65 years old. 65+, n = 65; 65−, n = 217. **(E, F)** Bar diagram summarizing the percentage of B cells **(E)** and CD4+ T cells **(F)** in the peripheral blood of NSCLC patients according to smoking history. Smokers, n = 103; non-smokers, n = 179. **(G)** Bar diagram summarizing the percentage of B cells in the peripheral blood of NSCLC patients according to cancer type. Data are shown as mean ± SEM; LUSC, lung squamous cell carcinoma, n = 39; LUAD, lung adenocarcinoma, n = 228. *p < 0.05; **p < 0.01; ****p < 0.0001.

We observed a higher proportion of B cells in female NSCLC patients than in male patients (**Figure 3A**). Furthermore, we found that the percentage of CD38+ CD45+ leukocytes and CD3+ T cells was higher in NSCLC patients under 65 years of age than in patients above 65 years of age (**Figures 3B, C**). But the percentage of NK cells was higher in patients above 65 years(**Figure 3D**). The percentage of B cells was higher in non-smokers, while the percentage of CD4+ T cells was higher in smokers (**Figures 3E, F**). Interestingly, the percentage of B cells in the PB of patients was higher in lung adenocarcinoma (LUAD) than in lung squamous cell carcinoma (LUSC) (**Figure 3G**). These results demonstrate that systemic immune dysregulation is correlated with the clinical features of NSCLC.

### Systemic Immune Dysregulation Correlates With the Non-Small Cell Lung Cancer Stage

**Figure 4:**
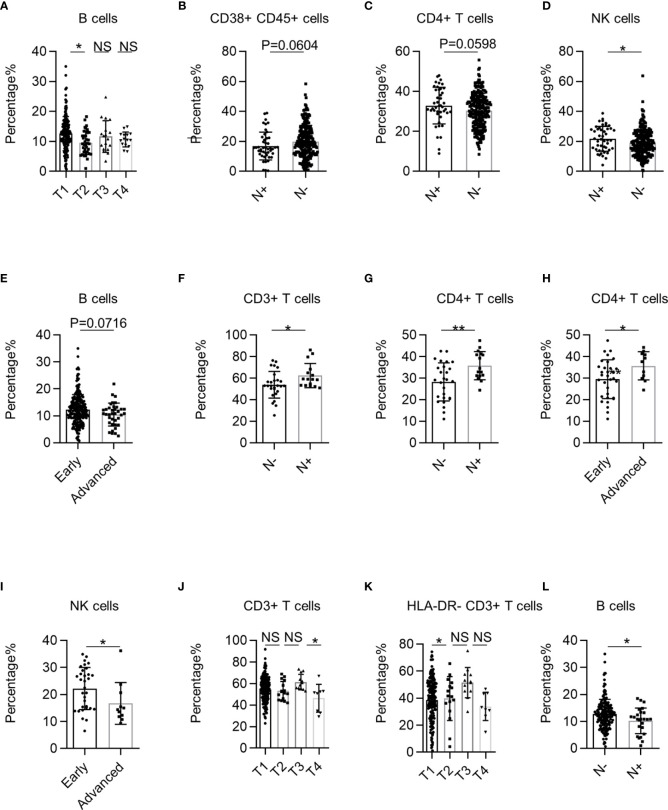
The correlation between systemic immune dysregulation and cancer stage. Systemic immune dysregulation in the validation group was analyzed to investigate the correlation between systemic immune dysregulation and the non-small cell lung cancer (NSCLC) stage. The TNM stage is based on the International Association for the Study of Lung Cancer (IASLC) cancer staging manual (8th version). **(A)** Bar diagram summarizing the percentage of B cells in the peripheral blood of NSCLC patients according to T stage. T1, n = 207; T2, n = 36; T3, n = 18; T4, n = 15. **(B–D)** Bar diagram summarizing the percentage of activated lymphocytes **(B)**, CD4+ T cells **(C)**, and NK cells **(D)** cells in the peripheral blood of NSCLC patients according to lymphatic metastasis. N+, lymphatic metastasis positive, n = 46; N−, lymphatic metastasis-negative, n = 235. **(E)** Bar diagram summarizing the percentage of B cells in the peripheral blood of NSCLC patients according to TNM stage. Early, TNM I+II stage, n = 242; Advanced, TNM III +IV stage, n = 38. **(F, G)** Bar diagram summarizing the percentage of CD3+ T cells **(F)** and CD4+ T cells **(G)** in the peripheral blood of lung squamous cell carcinoma (LUSC) patients according to lymphatic metastasis. N+, lymphatic metastasis positive, n = 16; N−, lymphatic metastasis-negative, n = 27. **(H, I)** Bar plot showing the percentage of CD4+ T cells **(H)** and NK cells **(I)** in the peripheral blood of LUSC patients according to TNM stage. Early, TNM I+II stage, n = 32; Advanced, TNM III +IV stage, n = 11. **(J, K)** Bar plot summarizing the percentage of CD3+ T cells **(J)** and resting T lymphocytes **(K)** in the peripheral blood of LUAD patients according to T stage. T1, n = 187; T2, n = 16; T3, n = 12; T4, n = 8. **(L)** Bar diagram summarizing the percentage of B cells in the peripheral blood of LUAD patients according to lymphatic metastasis. Data are shown as mean ± SEM; N+, lymphatic metastasis positive, n = 25; N−, lymphatic metastasis-negative, n = 198. NS, not statistically significant; *P < 0.05, **P < 0.01.

Next, we investigated the correlation between systemic immune dysregulation and T stage in NSCLC. The *p*-values for trend test between TNM stage and lymphocytes are summarized in **Supplementary Table 3**. The percentage of B cells was significantly higher in patients in the T1 stage than in the T2 stage (**Figure 4A**). Subsequently, we investigated the correlation between systemic immune dysregulation and N stage in NSCLC. The percentages of CD38+ CD45+ leukocytes (p = 0.0604) tended to be higher in the PB of patients without lymphatic metastasis (**Figure 4B**). Similarly, the percentage of CD4+ T cells (p = 0.0596) and NK cells tended to higher in patients with lymphatic metastasis (**Figures 4C, D**). We observed that the percentage of B cells (*p* = 0.0716) showed a downward trend in patients in the advanced TNM stage (**Figure 4E**).

We further analyzed the correlation between systemic immune dysregulation and the NSCLC TNM stage grouped by pathological subtype. First, we investigated the correlation between systemic immune dysregulation and the TNM stage of LUSC. The percentages of CD3+ and CD4+ T cells were significantly higher in LUSC patients with lymphatic metastasis (**Figures 4F, G**). Consistently, the percentages of CD4+ T cells were significantly higher in advanced-stage LUSC patients (**Figure 4H**). In contrast, the NK cell percentage was significantly lower in patients with advanced LUSC (**Figure 4I**). Next, we investigated the correlation between the systemic immune state and TNM stage of LUAD. The percentage of CD3+ T cells was significantly higher in patients with LUAD in the T3 stage than in the other stages (**Figure 4J**). Interestingly, the percentages of HLA-DR− CD3+ T cells were significantly higher in patients with LUAD at T3 than at T1 (**Figure 4K**). Regarding the N stage, we observed that the percentage of B cells was significantly lower in LUAD patients with lymphatic metastasis (**Figure 4L**). Together, these results demonstrate that systemic immune dysregulation is correlated with the NSCLC clinical stage.

### Systemic Immune Dysregulation Correlates With the Pathological Characteristics of Human Non-Small Cell Lung Cancer

**Figure 5:**
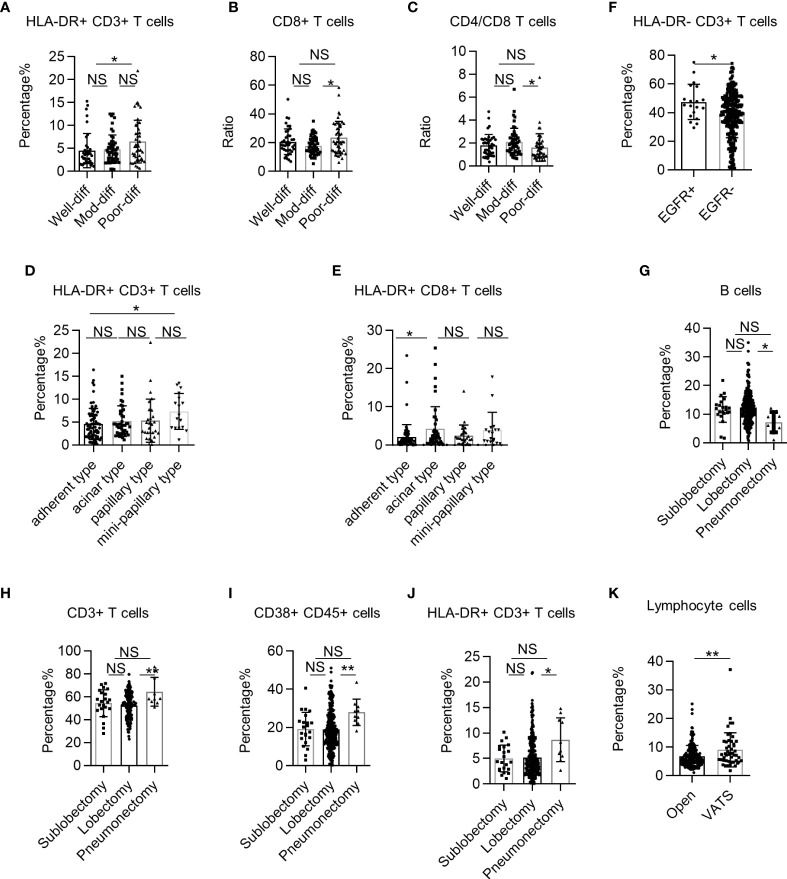
The correlation between systemic immune dysregulation and pathological characteristics or surgical procedure. Systemic immune dysregulation in the validation group was analyzed to investigate the correlation between systemic immune dysregulation and pathological characteristics or surgical procedures in non-small cell lung cancer (NSCLC) patients. **(A–C)** Bar diagram summarizing the percentage of HLA-DR+ CD3+ T cells **(A)**, CD8+ T cells **(B)**, and the CD4+ T cell/CD8+ T-cell ratio **(C)** in the peripheral blood of NSCLC patients according to tumor differentiation. Well differentiated, n = 40; moderately differentiated, n = 50; poorly differentiated, n = 47. **(D, E)** Bar diagram summarizing the percentage of HLA-DR+ CD3+ T cells **(D)** and HLA-DR+ CD8+ T cells **(E)** in the peripheral blood of LUAD patients according to histological classification. Adherent type, n = 81; acinar type, n = 44; papillary type, n = 29; mini-papillary type, n = 17. **(F)** Bar diagram summarizing the percentage of HLA-DR− CD3+ T cells in the peripheral blood of LUAD patients according to EGFR expression. EGFR+, n = 19; EGFR−, n = 262. **(G–J)** Bar diagram summarizing the percentage of B cells **(G)**, CD3+ T cells **(H)**, activated lymphocytes **(I)**, and HLA-DR+ CD3+ T cells **(J)** in the peripheral blood of NSCLC patients according to surgical excision extension. Sub-lobectomy, n = 22; lobectomy, n = 248; pneumonectomy, n = 10. **(K)** Bar diagram summarizing the percentage of total lymphocytes in the peripheral blood of NSCLC patients according to surgical procedure. Data are presented as mean ± SEM; open, n = 232; lobectomy, n = 49. NS, not statistically significant; *P < 0.05, **P < 0.01.

Pathological characteristics are closely associated with NSCLC treatment and prognosis (22). However, the correlation between pathological characteristics and systemic immune state in early human NSCLC remains unclear. Thus, we investigated the correlation between cancer cell differentiation and systemic immune state in NSCLC. We found that the percentages of HLA-DR+ CD3+ T cells and CD8+ T cells were significantly higher in NSCLC patients with poor cancer cell differentiation (**Figures 5A, B**). However, the CD4+ T cells/CD8+ T cell ratio was significantly lower in the PB of NSCLC patients with poor cancer cell differentiation (**Figure 5C**). Next, we analyzed the correlation between systemic immune state and histological classification of LUAD. We found that the adherent type had a lower percentage of HLA-DR+ CD3+ T cells and HLA-DR+ CD8+ T cells than the mini-papillary and acinar types, respectively (**Figures 5D, E**). We also observed significantly higher percentages of HLA-DR− CD3+ T cells in epidermal growth factor receptor (EGFR)+ LUAD patients (**Figure 5F**). These findings demonstrate that systemic immune dysregulation in patients is correlated with pathological NSCLC characteristics.

### Systemic Immune State Correlates With Surgical Procedures in Non-Small Cell Lung Cancer

Radical surgery is the most important treatment for early NSCLC. However, how the surgical procedure influences the systemic immune state of NSCLC is unknown. First, we investigated the correlation between surgical excision extension and systemic immune state for NSCLC. We found that the percentage of B cells in the PB of NSCLC patients was lower in the pneumonectomy group than in the lobectomy group (**Figure 5G**). In contrast, the percentages of multiple cell subsets, such as CD3+ T cells, CD38+ CD45+ lymphocytes, and HLA-DR+ CD3+ T cells, in the PB of NSCLC patients were higher in the pneumonectomy group than in the lobectomy or sub-lobectomy groups (**Figures 5H–J**). Next, we investigated the impact of minimally invasive surgery on the systemic immune state of NSCLC without regard to excision extension. The percentage of total lymphocytes in NSCLC patients was higher in the video-assisted thoracic surgery (VATS) group than in the open group (**Figure 5K**). These findings suggest that extended trauma induced by large surgical excision extension may aggravate systemic immune dysregulation in patients with locally progressive NSCLC.

### Systemic Immune Dysregulation Correlates With Complications and Systemic Inflammatory Response Syndrome

**Figure 6:**
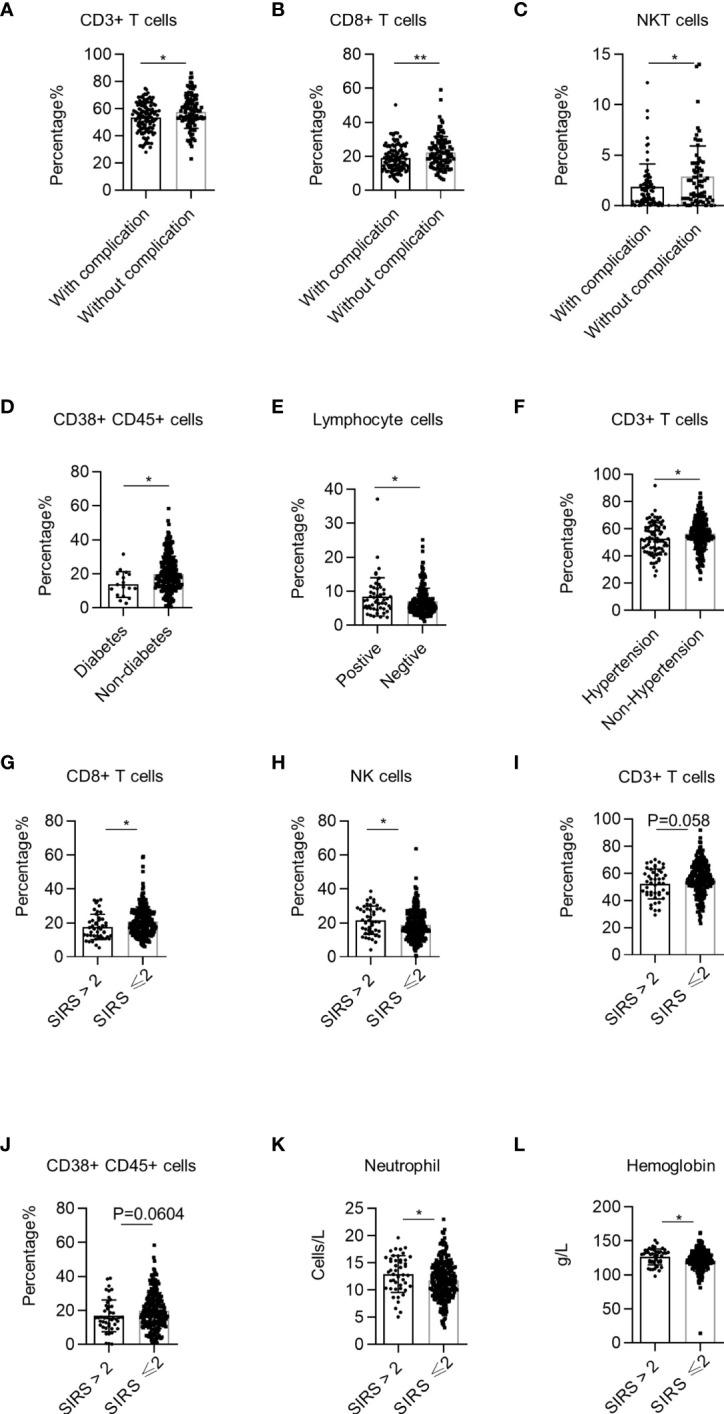
Correlation between systemic immune dysregulation and surgical complication or perioperative systemic inflammatory response syndrome (SIRS). Systemic immune dysregulation in the validation group was analyzed to investigate the correlation between systemic immune dysregulation and surgical complications or perioperative SIRS in non-small cell lung cancer (NSCLC) patients. Perioperative SIRS was defined as a SIRS score of >2. **(A–C)** The percentage of CD3+ T cells **(A)**, CD8+ T cells **(B)**, and NKT cells **(C)** in the peripheral blood of NSCLC patients according to surgical complication. With complications, n = 125; without complications, n = 103. **(D)** Percentage of activated lymphocytes in the peripheral blood of NSCLC patients according to complications of diabetes. Diabetes, n = 17; without diabetes, n = 263. **(E)** Percentage of total lymphocytes in the peripheral blood of NSCLC patients with pulmonary infection. Sputum culture positive, n = 51; sputum culture negative, n = 231. **(F)** Percentage of CD3+ T cells in the peripheral blood of NSCLC patients with hypertension. Hypertension, n = 76; non-hypertension, n = 206; *p < 0.05. **(G–J)** Percentage of CD8+ T cells **(G)**, NK cells **(H)**, CD3+ T cells **(I)**, and activated lymphocytes **(J)** in the peripheral blood of NSCLC patients according to SIRS score. SIRS score >2, n = 46; SIRS score ≤2, n = 235. **(K)** Neutrophil count in the peripheral blood of NSCLC patients according to SIRS score. SIRS score >2, n = 47; SIRS score ≤2, n = 245. **(L)** Hemoglobin quantity in the peripheral blood of NSCLC patients according to the SIRS score. Data are shown as mean ± SEM; SIRS score >2, n = 47; SIRS score ≤2, n = 237. *P < 0.05, **P < 0.01.

Complications and systemic inflammatory response syndrome (SIRS) are important risk factors that cause perioperative death in NSCLC patients undergoing surgery. NSCLC patients with complications had lower percentages of CD3+ T cells, CD8+ T cells, and NKT cells than patients without complications (**Figures 6A–C**). Specifically, NSCLC patients with diabetes had lower percentages of CD38+ CD45+ lymphocytes than patients without diabetes (**Figure 6D**). The percentage of total lymphocytes in NSCLC patients with positive bacterial sputum cultures was higher than that of patients with negative cultures (**Figure 6E**). Additionally, the CD3+ T cell percentages in patients with hypertension were lower than in patients without hypertension (**Figure 6F**). These findings demonstrate that systemic immune dysregulation is correlated with NSCLC complications. Next, we investigated the correlation between the perioperative systemic immune state of NSCLC and SIRS using the previously reported definition of SIRS (23). We found that the percentages of CD8+ T cells and NK cells in the PB of NSCLC patients with SIRS were significantly lower than those in patients without SIRS (**Figures 6G, H**). There was also a tendency for lower percentages of CD3+ T cells (*p* = 0.0585) and CD38+ CD45+ lymphocytes (*p* = 0.0604) (**Figures 6I, J**). Using the data from routine blood tests of NSCLC patients, we found that neutrophils and hemoglobin levels of NSCLC patients were also correlated with SIRS (**Figures 6K, L**). These findings demonstrate that systemic immune dysregulation in NSCLC patients is correlated with the risk of perioperative SIRS.

## Discussion

Immune surveillance plays a crucial role in the development of lung cancer (3). Unfortunately, with the development of lung cancer, cancer cell immunogenicity can be lost, resulting in immune escape and cancer progression (24). Targeting the immune escape of cancer using novel immunotherapies, such as immune checkpoint blockade, is encouraging in treating advanced NSCLC stages (25). However, the survival benefits obtained from immune checkpoint blockade are still not satisfactory (26). Accumulating evidence shows a higher response ratio of immune checkpoint blockade in resectable NSCLC, suggesting that a better therapeutic effect may be acquired in relatively early NSCLC stages (27). However, the details of local and systematic immune perturbations in human NSCLC are unclear.

A recent study showed that immune perturbation in the tumor microenvironment occurs in the initial phase of human lung squamous cancer (5). Analogously, an innate immune perturbation in the tumor microenvironment was also observed in early human LUAD (6). However, whether systematic immune perturbations occur in early NSCLC remains unknown. To uncover the systemic immune state of early NSCLC and its potential correlations with clinical features, we used a panel of nine markers to profile 11 leukocyte subsets in the PB in a large cohort of 326 early NSCLC patients.

Our results revealed remarkable systemic immune dysregulation in early NSCLC characterized by a reduction in total lymphocytes, CD3+ T cells, HLA-DR− CD3+ T cells, CD4+ T cells, and NKT cells. However, several lymphocyte subsets such as CD38+ lymphocytes and HLA-DR+ CD3+ T cells were increased. Since radical surgery is among the most important treatments for early NSCLC, we profiled the leukocyte subsets in paired PB of 29 patients with early NSCLC and found that surgery further decreased the percentage of total lymphocytes, CD3+ T cells, HLA-DR− CD3+ T cells, and CD4+ T cells in the PB of postsurgery patients. However, CD38+ lymphocyte cells, B cells, and HLA-DR+ CD8+ T cells increased after surgery. We found that the percentage and absolute counts of CD45+ lymphocytes decreased. Many studies demonstrated that non-responding patients show more PD-1+ CD38+ CD8+ cells in tumors and blood than responders for PD-1 targeted therapy (28). CD38^low^ T cells exhibit enhanced oxidative phosphorylation and significantly improved tumor control (29, 30). Decreased total lymphocytes and high CD38 expression in the PB of early NSCLC patients may represent a state of immunosuppression. Consistent with a previous study, our findings further uncover the details of systemic immune dysregulation in early NSCLC (21).

Accumulating evidence shows a correlation between peripheral leukocyte subsets and the outcome of advanced NSCLC (15–17, 19). However, there are few studies on the correlation between peripheral regulatory T cells and the clinical features of early NSCLC (18). An increasing number of studies show that peripheral leukocyte subsets are correlated with therapeutic effects and toxicity of immune checkpoint inhibitors in advanced NSCLC (31–36). A recent study reported that a decrease in peripheral CD4+ T cell clones is correlated with disease progression in advanced NSCLC patients treated with PD-1 inhibitors (37). These findings suggest that the systemic immune state of NSCLC plays a crucial role in cancer development and treatment. As mentioned before, a higher pathological response rate in neoadjuvant therapy also underlines the significance of the systemic immune state of patients treated with immune checkpoint blockade of early NSCLC (27, 38). In our current study, we found that the systemic immune state of NSCLC patients is correlated with clinical features such as age, sex, smoking history, body weight, and pathological types. These features also correlate with immune checkpoint blockade in early NSCLC (27). We also observed a correlation between the systemic immune state of patients and tumor stage, differentiation, pathological characteristics, surgical procedures, complications, and perioperative SIRS, which are closely linked to NSCLC treatment and prognosis (3).

Our study has some limitations. Due to the limited clinical samples, we could not conduct large cohort studies on NSCLC patients. In addition, we cannot completely rule out that the systemic immune dysregulation was not a product of surgically induced inflammation. A larger cohort study is necessary to further confirm these results.

In conclusion, our study uncovered several dramatic systemic immune dysregulation in early NSCLC. Moreover, we observed that systemic immune dysregulation in NSCLC patients correlates with clinical features that are closely related to NSCLC treatment and prognosis. Therefore, detecting systemic immune dysregulation in the PB of patients is a promising predictive biomarker for the prognosis and treatment of early NSCLC. Further investigation of the underlying mechanisms of systemic immune dysregulation will help to develop new therapeutic strategies for early NSCLC.

## Data Availability Statement

The original contributions presented in the study are included in the article/**Supplementary Material**. Further inquiries can be directed to the corresponding authors.

## Ethics Statement

The studies involving human participants were reviewed and approved by The Review Board of the Second Affiliated Hospital of Zhejiang University School of Medicine. The patients/participants provided their written informed consent to participate in this study.

## Author Contributions

ZXH, MJL, FD, YYC, ZWX, and PW performed the experiments. ZXH, MJL, FD, YMW, ZYZ, WSL, DW, and PW collected the samples and the clinical data. ZXH, MJL, SHJ, ZWX, and PW analyzed the data. ZXH, MJL, SHJ, ZWX, and PW designed the study, interpreted the data, and wrote the manuscript. SHJ and PW supervised the project. All authors contributed to the article and approved the submitted version.

## Funding

This work was supported by the National Natural Science Foundation of China (81572800 and 82073141 to PW, and 82073142 to DW), the Fundamental Research Funds for the Central Universities (2019QNA7025 to PW), and the Natural Science Foundation of Zhejiang Province (LR22H160006 and LY15H160041 to PW, and LY19H160050 to DW).

## Conflict of Interest

The authors declare that the research was conducted in the absence of any commercial or financial relationships that could be construed as a potential conflict of interest.

## Publisher’s Note

All claims expressed in this article are solely those of the authors and do not necessarily represent those of their affiliated organizations, or those of the publisher, the editors and the reviewers. Any product that may be evaluated in this article, or claim that may be made by its manufacturer, is not guaranteed or endorsed by the publisher.

## Supplementary Material

Supplementary Figure 1 Systemic immune disturbances in early NSCLC. Systemic immune disturbances were assessed by flow cytometry on single cell suspensions prepared from the peripheral blood of NSCLC patients and healthy donors. Data are shown as mean ± SEM; NSCLC patients, n=34; healthy donors, n=34; NS, not statistically significant; *P < 0.05, **P < 0.01, ***P < 0.001, ****P < 0.0001. A-F. Bar plots summarizing the absolute counts of total lymphocytes **(A)**, activated lymphocytes **(B)**, CD3+ T cells **(C)**, NKT cells **(D)**, quiescent CD3+ T cells **(E)**, CD4+T cells **(F)** in the peripheral blood of NSCLC patients and healthy donors.

Supplementary Figure 2 Systemic immune disturbances of postsurgical NSCLC patients in the discovery group. Systemic immune disturbances were assessed by flow cytometry on single cell suspensions prepared from the peripheral blood of post-operative NSCLC patients and healthy donors. Data are shown as mean ± SEM; NSCLC patients, n=29; healthy donors, n=34; NS, not statistically significant; *P < 0.05, **P < 0.01, ***P < 0.001, ****P < 0.0001. A-K. Bar plots summarizing the percentage of total lymphocytes **(A)**, activated lymphocyte s **(B)**, CD3+ T cells **(C)**, NKT cells **(D)**, CD4+ T cells **(E)**, NK cells **(F)**, B cells **(G)**, HLA-DR+CD3+T cells **(H)**, HLA-DR-CD3+T cells **(I)**, CD8+ T cells **(J)**, and HLA-DR+CD8+T cells **(K)** in the peripheral blood of NSCLC patients and healthy donors. L. Bar plot summarizing the CD4+ T cell/CD8+ T cell ratio in the peripheral blood of NSCLC patients and healthy donors.

Supplementary Figure 3 Impact of surgery on systemic immune disturbances of early NSCLC. Systemic immune disturbances were assessed by flow cytometry on single cell suspensions prepared from peripheral blood of paired samples from NSCLC patients pre- and post-operatively. Data are shown as mean ± SEM; preoperative patients n=29; post-operation patients n=29; NS: not statistically significant, *P < 0.05, **P < 0.01, ***P < 0.001, ****P < 0.0001. A-I. Bar plots summarizing the absolute counts of total lymphocytes **(A)**, CD3+ T cells **(B)**, activated lymphocytes **(C)**, CD4+T cells **(D)**, CD4/CD8+ T cells **(E)**, NKT cells **(F)**, quiescent and activated CD3+ T cells **(G)**, B cells **(H)**, and HLA-DR+CD8+T cells **(I)** in the peripheral blood of NSCLC patients pre- and post-surgery.

Supplementary Figure 4 The correlation between systemic immune disturbances and surgical procedure in the discovery group. Systemic immune disturbances in the discovery group were analyzed to investigate the correlation between systemic immune disturbances and surgical procedures in NSCLC patients. Data are shown as mean ± SEM; sublobectomy, n=24; lobectomy, n=5; NS, not statistically significant. A-K. Bar plots summarizing the percentage of total lymphocytes **(A)**, activated lymphocytes **(B)**, CD3+ T cells **(C)**, NKT cells **(D)**, CD4+ T cells **(E)**, NK cells **(F)**, B cells **(G)**, HLA-DR+CD3+T cells **(H)**, HLA-DR-CD3+T cells **(I)**, CD8+ T cells **(J)**, and HLA-DR+CD8+T cells **(K)** in the peripheral blood of NSCLC patients with surgical excision extension. L. Bar plot summarizing the CD4+ T cells/CD8+ T cell ratio in the peripheral blood of NSCLC patients with surgical excision extension.

Supplementary Figure 5 Systemic immune disturbances of post-operation NSCLC patients in the validation group. Systemic immune disturbances were assessed by flow cytometry on single cell suspensions prepared from the peripheral blood of post-operation NSCLC patients and healthy donors. Data are shown as mean ± SEM; NSCLC patients n=292; healthy donors n=34; NS: not statistically significant, *P < 0.05, **P < 0.01, ***P < 0.001, ****P < 0.0001. A-K. Bar plots summarizing the percentage of total lymphocytes **(A)**, activated lymphocytes **(B)**, CD3+ T cells **(C)**, NKT cells **(D)**, CD4+ T cells **(E)**, NK cells **(F)**, B cells **(G)**, HLA-DR+CD3+T cells **(H)**, HLA-DR-CD3+T cells **(I)**, CD8+ T cells **(J)**, and HLA-DR+CD8+T cells **(K)** in the peripheral blood of NSCLC patients and healthy donors. L. Bar plot summarizing the CD4+ T cell/CD8+ T cell ratio in the peripheral blood of NSCLC patients and healthy donors.
