# Supplementary figures and images for: Systemic Immune Dysregulation Correlates With Clinical Features of Early Non-Small Cell Lung Cancer
Source: Front Immunol. 2022 Jan 18;12:754138. doi: 10.3389/fimmu.2021.754138 (PMC8804248; doi:10.3389/fimmu.2021.754138)

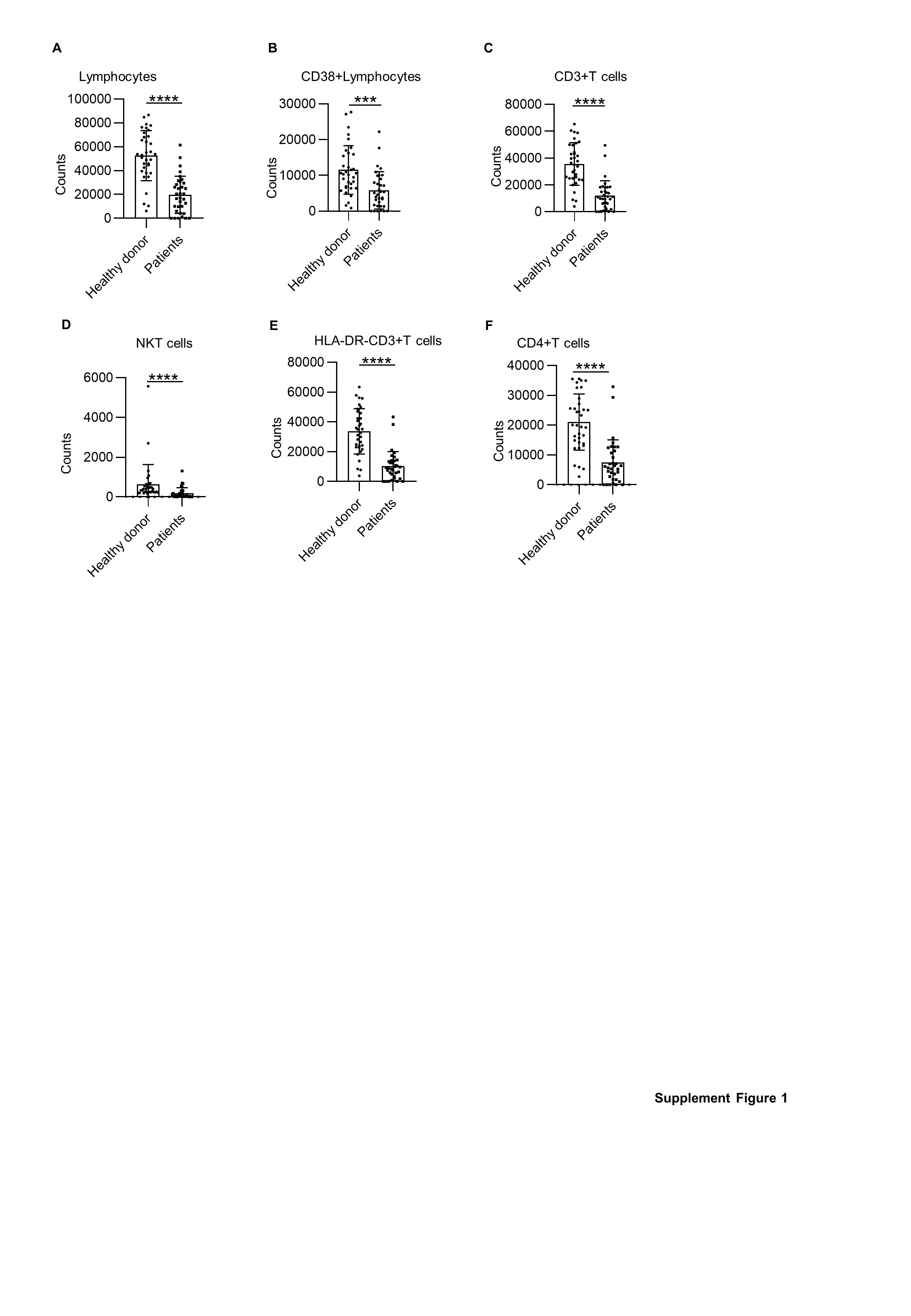

Supplement: Supplementary Figure 1 — Systemic immune disturbances in early NSCLC. Systemic immune disturbances were assessed by flow cytometry on single cell suspensions prepared from the peripheral blood of NSCLC patients and healthy donors. Data are shown as mean ± SEM; NSCLC patients, n=34; healthy donors, n=34; NS, not statistically significant; *P < 0.05, **P < 0.01, ***P < 0.001, ****P < 0.0001. A-F. Bar plots summarizing the absolute counts of total lymphocytes (A), activated lymphocytes (B), CD3+ T cells (C), NKT cells (D), quiescent CD3+ T cells (E), CD4+T cells (F) in the peripheral blood of NSCLC patients and healthy donors. [file Image_1.tif]

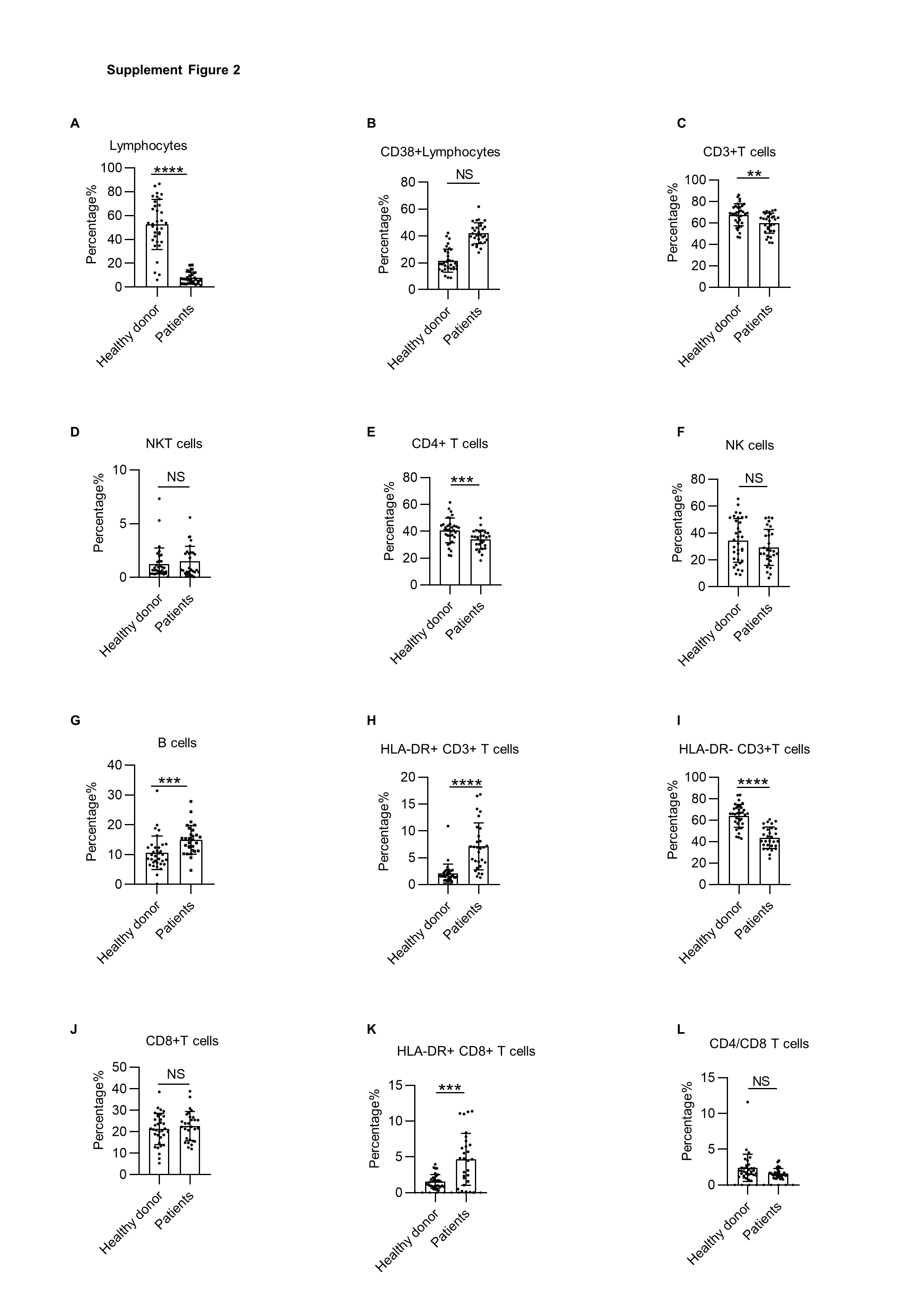

Supplement: Supplementary Figure 2 — Systemic immune disturbances of postsurgical NSCLC patients in the discovery group. Systemic immune disturbances were assessed by flow cytometry on single cell suspensions prepared from the peripheral blood of post-operative NSCLC patients and healthy donors. Data are shown as mean ± SEM; NSCLC patients, n=29; healthy donors, n=34; NS, not statistically significant; *P < 0.05, **P < 0.01, ***P < 0.001, ****P < 0.0001. A-K. Bar plots summarizing the percentage of total lymphocytes (A), activated lymphocyte s (B), CD3+ T cells (C), NKT cells (D), CD4+ T cells (E), NK cells (F), B cells (G), HLA-DR+CD3+T cells (H), HLA-DR-CD3+T cells (I), CD8+ T cells (J), and HLA-DR+CD8+T cells (K) in the peripheral blood of NSCLC patients and healthy donors. L. Bar plot summarizing the CD4+ T cell/CD8+ T cell ratio in the peripheral blood of NSCLC patients and healthy donors. [file Image_2.tif]

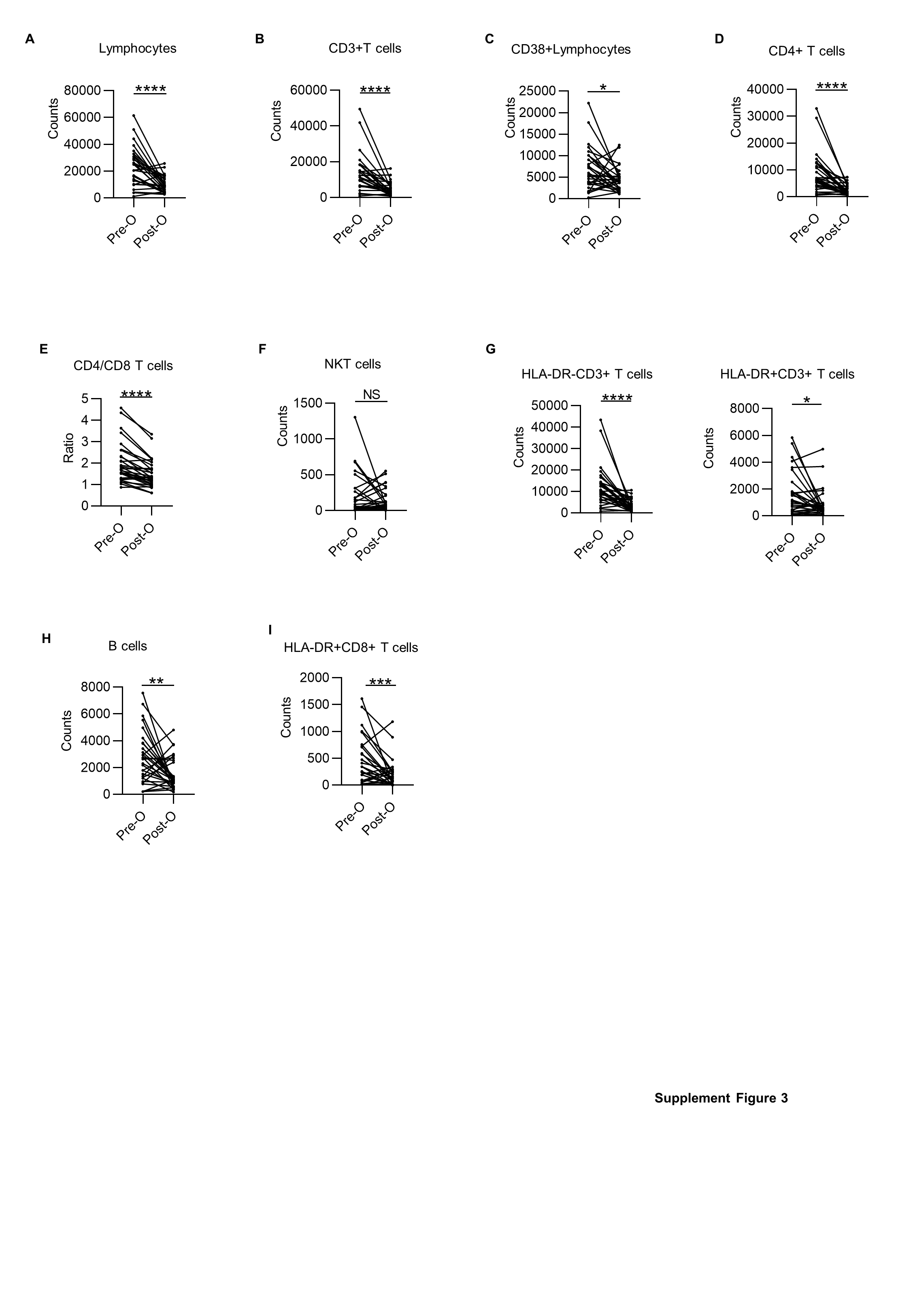

Supplement: Supplementary Figure 3 — Impact of surgery on systemic immune disturbances of early NSCLC. Systemic immune disturbances were assessed by flow cytometry on single cell suspensions prepared from peripheral blood of paired samples from NSCLC patients pre- and post-operatively. Data are shown as mean ± SEM; preoperative patients n=29; post-operation patients n=29; NS: not statistically significant, *P < 0.05, **P < 0.01, ***P < 0.001, ****P < 0.0001. A-I. Bar plots summarizing the absolute counts of total lymphocytes (A), CD3+ T cells (B), activated lymphocytes (C), CD4+T cells (D), CD4/CD8+ T cells (E), NKT cells (F), quiescent and activated CD3+ T cells (G), B cells (H), and HLA-DR+CD8+T cells (I) in the peripheral blood of NSCLC patients pre- and post-surgery. [file Image_3.tif]

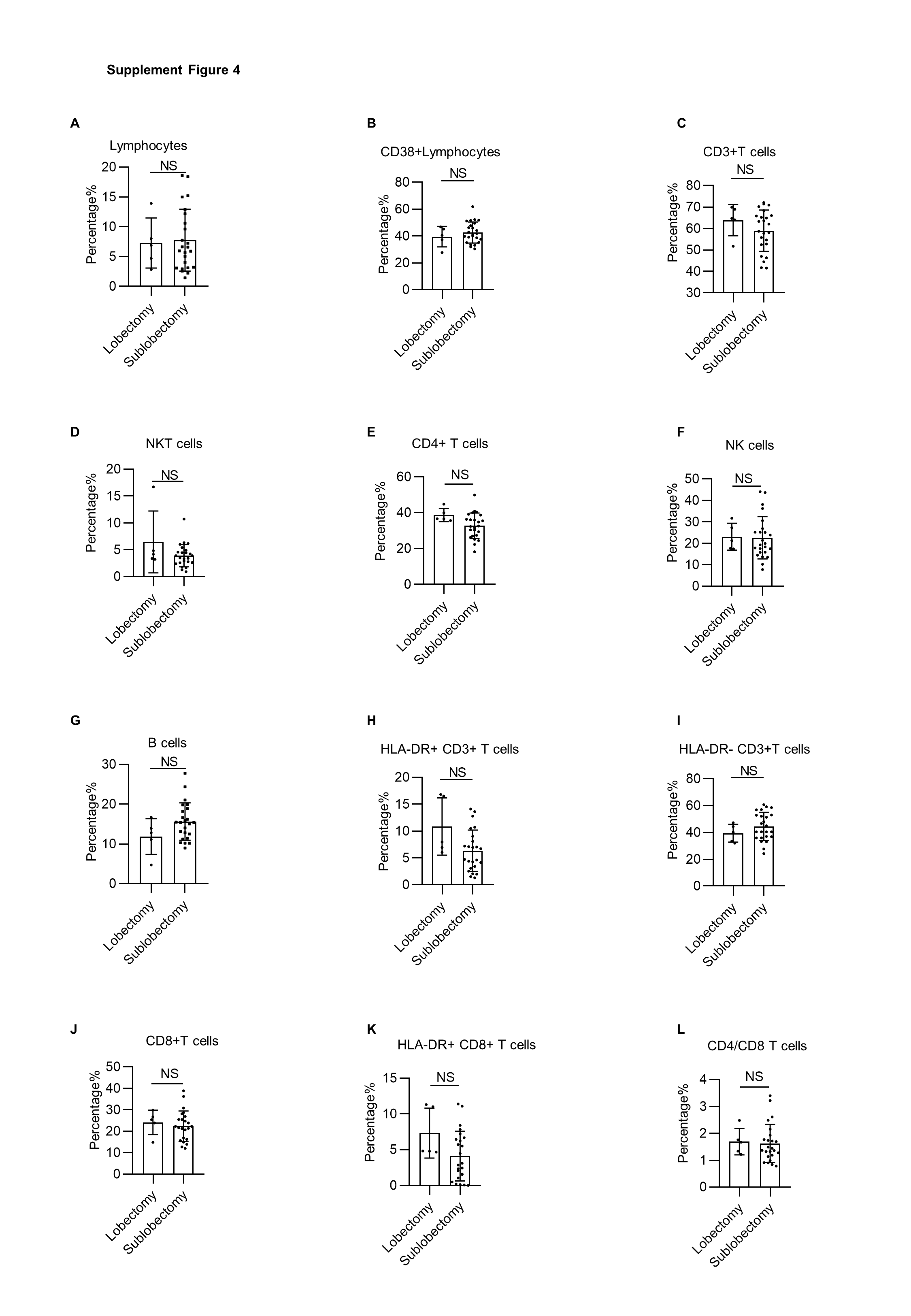

Supplement: Supplementary Figure 4 — The correlation between systemic immune disturbances and surgical procedure in the discovery group. Systemic immune disturbances in the discovery group were analyzed to investigate the correlation between systemic immune disturbances and surgical procedures in NSCLC patients. Data are shown as mean ± SEM; sublobectomy, n=24; lobectomy, n=5; NS, not statistically significant. A-K. Bar plots summarizing the percentage of total lymphocytes (A), activated lymphocytes (B), CD3+ T cells (C), NKT cells (D), CD4+ T cells (E), NK cells (F), B cells (G), HLA-DR+CD3+T cells (H), HLA-DR-CD3+T cells (I), CD8+ T cells (J), and HLA-DR+CD8+T cells (K) in the peripheral blood of NSCLC patients with surgical excision extension. L. Bar plot summarizing the CD4+ T cells/CD8+ T cell ratio in the peripheral blood of NSCLC patients with surgical excision extension. [file Image_4.tif]

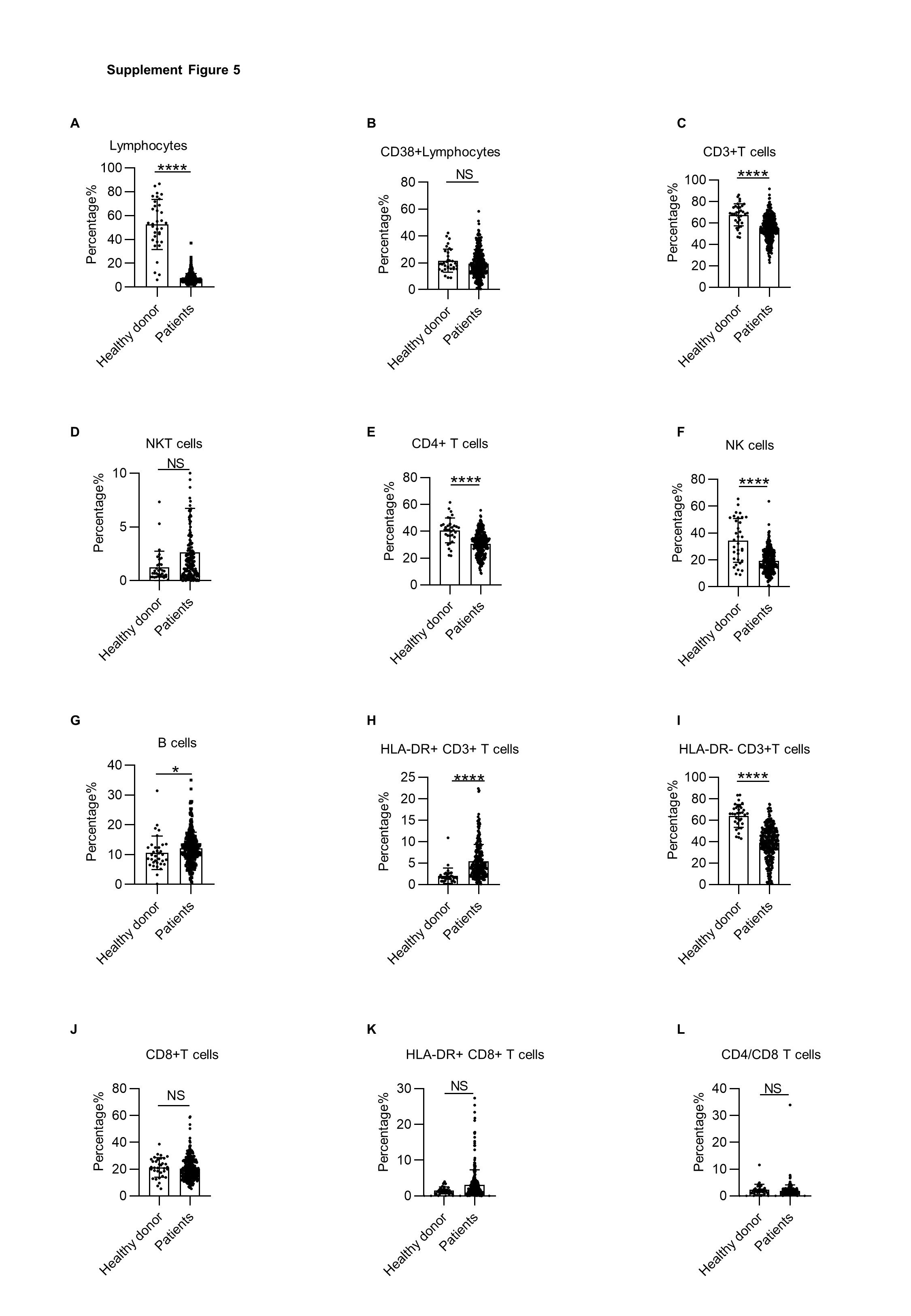

Supplement: Supplementary Figure 5 — Systemic immune disturbances of post-operation NSCLC patients in the validation group. Systemic immune disturbances were assessed by flow cytometry on single cell suspensions prepared from the peripheral blood of post-operation NSCLC patients and healthy donors. Data are shown as mean ± SEM; NSCLC patients n=292; healthy donors n=34; NS: not statistically significant, *P < 0.05, **P < 0.01, ***P < 0.001, ****P < 0.0001. A-K. Bar plots summarizing the percentage of total lymphocytes (A), activated lymphocytes (B), CD3+ T cells (C), NKT cells (D), CD4+ T cells (E), NK cells (F), B cells (G), HLA-DR+CD3+T cells (H), HLA-DR-CD3+T cells (I), CD8+ T cells (J), and HLA-DR+CD8+T cells (K) in the peripheral blood of NSCLC patients and healthy donors. L. Bar plot summarizing the CD4+ T cell/CD8+ T cell ratio in the peripheral blood of NSCLC patients and healthy donors. [file Image_5.tif]
