# Supplementary material for: Systemic Immune Dysregulation Correlates With Clinical Features of Early Non-Small Cell Lung Cancer
Source: Front Immunol. 2022 Jan 18;12:754138. doi: 10.3389/fimmu.2021.754138 (PMC8804248; doi:10.3389/fimmu.2021.754138)
Supplement: Supplementary file 6 [file Table_1.docx]

**Table 1** Characteristics of cancer patients and healthy donor.

| **Characteristics** | **Discovery group** | **Validation group** | **Healthy Donor** |
| --- | --- | --- | --- |
|  | n=34 | n=292 | n=34 |
| Age, year(mean) | 57.4(67) | 58.9(50) | 57.4(61) |
| Male, n (%) | 11(32.4) | 137(46.9) | 11(32.4) |
| Smoking, n (%) | 7(20.6) | 107(36.6) | 7(20.6) |
| Hypertension, n (%) | 5(14.7) | 77(26.4) | 8(23.5) |
| Diabetes, n (%) | 2(5.9) | 18(6.2) | 1(2.9) |
| Cardiopulmonary disease, n (%) | 0(0) | 10(3.4) | 1(2.9) |
| TNM stage, n (%) |  |  |  |
| I | 25(73.5) | 216(74.0) | N/A |
| II | 0(0.0) | 35(12.0) | N/A |
| III  IV | 3(8.3)  0(0.0) | 37(12.7)  4(1.4) | N/A  N/A |
| Histological type, n (%) |  |  |  |
| LUAD | 32(94.1) | 230(78.8) | N/A |
| LUSC  Others | 1(2.9)  1(2.9) | 44(15.1)  18(6.2) | N/A  N/A |

TNM stage is according to IASLC cancer staging manual (8th version).

Abbreviations: LUAD: lung adenocarcinoma; LUSC: squamous cell lung carcinoma; N/A, not applicable;
