# Supplementary material for: Systemic Immune Dysregulation Correlates With Clinical Features of Early Non-Small Cell Lung Cancer
Source: Front Immunol. 2022 Jan 18;12:754138. doi: 10.3389/fimmu.2021.754138 (PMC8804248; doi:10.3389/fimmu.2021.754138)
Supplement: Supplementary file 7 [file Table_2.docx]

**Table 2** Immunophenotyping analysis for cancer patients and normal subjects.

| **Parameter** | **Discovery group** | **Validation group** | **Healthy Donor** |
| --- | --- | --- | --- |
| Number | n=34 | n=292 | n=34 |
| Total lymphocytes  Activated lymphocytes  T lymphocytes  B lymphocytes | 18.5(0.71-49)  34.1(18.5-62.6)  63.7(37.7-83.4)  11.4(2.78-20.9) | 7.19(1.1-37.1)  19.5(0.6-58.4)  55.4(23.1-91.9)  12.1(0.7-35) | 52.6(6.24-86.7)  21.6(8.78-42.3)  67.59(46.5-86.1)  10.61(0.15-31.4) |
| NK cells | 23.6(5.59-56) | 19.3(0.4-63.6) | 34.5(8.91-65.4) |
| NKT cells | 0.92(0.02-6.3) | 2.7(0-36) | 1.25(0.09-7.33) |
| T helper cells | 38.5(22.1-67.5) | 30.7(8.5-55.7) | 40.65(21.7-61.6) |
| T cytotoxic cells | 20.9(6.82-35.7) | 20.3(5.3-59.1) | 21.26(5.31-38.6) |
| Activated T lymphocytes | 4.62(0.55-19.4) | 5.39(0.1-22.4) | 2.62(0.61-10.9) |
| Resting T lymphocytes | 54.8(34.8-78.6) | 39(1.1-75.4) | 64.03(42.8-83.6) |
| Activated T cytotoxic cells | 2.37(0.0-14.0) | 3.14(0-27.4) | 1.57(0.3-3.98) |
| CD4/CD8 | 2.18(0.89-4.88) | 1.79(0.29-7.73) | 2.40(0.57-11.6) |

Values indicate average and range for the percentage of lymphocyte subsets. Total lymphocytes (CD45+ lymphocytes); Activated lymphocytes (CD38+ lymphocytes); T lymphocytes (CD3+ T lymphocytes); B lymphocytes (CD19+ B lymphocytes); NK cells (CD16+ CD56+ CD3- lymphocytes); NKT cells (CD16+ CD56+ CD3+ lymphocytes); T helper cells (CD4+ CD3+ T lymphocytes); T cytotoxic cells (CD8+ CD3+ T lymphocytes); Activated T lymphocytes (HLA-DR+ CD3+ T lymphocytes); Resting T lymphocytes (HLA-DR- CD3+ T lymphocytes); Activated T cytotoxic cells (HLA-DR+ CD8+ T lymphocytes).
