# Supplementary material for: Systemic Immune Dysregulation Correlates With Clinical Features of Early Non-Small Cell Lung Cancer
Source: Front Immunol. 2022 Jan 18;12:754138. doi: 10.3389/fimmu.2021.754138 (PMC8804248; doi:10.3389/fimmu.2021.754138)
Supplement: Supplementary file 8 [file Table_3.docx]

**Table 3** Spearman’s rank correlation coefficient.

| P value for trend | | | |
| --- | --- | --- | --- |
| Subsets | TNM stage | T stage | N stage |
| Total lymphocytes  Activated lymphocytes  T lymphocytes  B lymphocytes | 0.0334  0.3752  0.0309  0.0017 | 0.0553  0.4243  0.0912  0.0019 | 0.1508  0.5009  0.0488  0.0041 |
| NK cells | 0.4663 | 0.8957 | 0.6185 |
| NKT cells | 0.7793 | 0.8651 | 0.7141 |
| T helper cells | 0.0908 | 0.7409 | 0.0579 |
| T cytotoxic cells | 0.0806 | 0.1057 | 0.0835 |
| Activated T lymphocytes | 0.0334 | 0.0042 | 0.2902 |
| Resting T lymphocytes | 0.3967 | 0.1507 | 0.7429 |
| Activated T cytotoxic cells | 0.1832 | 0.0954 | 0.7748 |

Total lymphocytes (CD45+ lymphocytes); Activated lymphocytes (CD38+ lymphocytes); T lymphocytes (CD3+ T lymphocytes); B lymphocytes (CD19+ B lymphocytes); NK cells (CD16+ CD56+ CD3- lymphocytes); NKT cells (CD16+ CD56+ CD3+ lymphocytes); T helper cells (CD4+ CD3+ T lymphocytes); T cytotoxic cells (CD8+ CD3+ T lymphocytes); Activated T lymphocytes (HLA-DR+ CD3+ T lymphocytes); Resting T lymphocytes (HLA-DR- CD3+ T lymphocytes); Activated T cytotoxic cells (HLA-DR+ CD8+ T lymphocytes).
